# Speckle tracking imaging evaluation of left ventricular myocardial work comparing right ventricular septal pacing with His-Purkinje system area pacing

**DOI:** 10.3389/fcvm.2022.949841

**Published:** 2022-10-25

**Authors:** Qingguo Meng, Yao Li, Sijia Wang, Tianhang Feng, Huijun Xu, Juan Liu, Xuebing Liu, Zhiyu Guo, Yan Deng, Chunmei Li, Yijia Tang, Lixue Yin

**Affiliations:** ^1^Ultrasound in Cardiac Electrophysiology and Biomechanics Key Laboratory of Sichuan Province, Sichuan Provincial People’s Hospital, University of Electronic Science and Technology of China, Chengdu, China; ^2^Department of Cardiac Function, Chengdu First People’s Hospital, Chengdu, China; ^3^Sichuan Provincial People’s Hospital, University of Electronic Science and Technology of China, Chengdu, China; ^4^Department of Gerontology, Xiqing Hospital, Tianjin, China; ^5^Chengdu Women’s and Children’s Central Hospital, Chengdu, China

**Keywords:** myocardial work, right ventricular septal pacing, His-Purkinje system area pacing, constructive work, work efficiency

## Abstract

**Aims:**

We sought to objectively assess left ventricular myocardial work (MW) parameters after right ventricular septal pacing (VSP) and His-Purkinje system area pacing (HPSAP) procedures.

**Materials and methods:**

Patients undergoing double-chamber pacemaker implantation for III-degree atrioventricular block (III° AVB) were assessed 1 year after implantation. VSP and HPSAP groups (20 and 23 patients, respectively) were compared against 40 healthy age-matched volunteers. Two-dimensional ultrasound speckle tracking imaging was used to obtain the global myocardial work index (GWI), global myocardial work efficiency (GWE), global myocardial constructive work (GCW), global myocardial wasted work (GWW), left ventricular stratified strain, and peak strain dispersion (PSD).

**Results:**

GWI, GWE, and GCW parameters were improved in HPSAP compared to VSP, while GWW was significantly larger in the VSP group compared to the HPSAP group (all *p* < 0.05). HPSAP outperformed the VSP group in comparisons of global left ventricular longitudinal strain and stratified strain. Compared to controls, the GCW of all segmental myocardium (17/17 segments) in the VSP group was significantly reduced, while 70.59% (12/17 segments) in the HPSAP group was lower than the control group. GCW in the left ventricular segment of the HPSAP group was bigger than the VSP group (29.41%; 5/17 segments) and mainly concentrated in the ventricular septum and inferior wall.

**Conclusion:**

Our findings suggest that HPSAP performance outcomes are improved over VSP after 1 year, especially in left ventricular contractile synchrony, and HPSAP is beneficial to the effective myocardial work of the left ventricle.

## Background

Previous studies have shown that different cardiac pacing methods can lead to reduced left ventricular systolic and diastolic function (1). With the advent of active helical electrodes, cardiac electrophysiologists are increasingly looking for ways to improve physiological cardiac pacing in order to change the structural remodeling and abnormal changes in cardiac function in affected patients. At present, the main clinical techniques used for physiological cardiac pacing involve right ventricular septal pacing (VSP) and His-Purkinje system area pacing (HPSAP). However, it remains unclear which of the two methods provides the best improvement in cardiac function. Some studies showed that a high long-term proportion of right ventricular pacing increased the risk of atrial fibrillation and heart failure (2). Compared with His bundle pacing, the HPSAP could cross below or more distal to the block site of His bundle pacing, which had a higher threshold perception (3). However, there were few relevant clinical studies, which necessitated more clinical research findings (4). Nevertheless, to optimize the patient’s cardiac functional state after cardiac pacing, it is important to examine the differences between these two cardiac pacing methods and understand the pathophysiological mechanism underlying any differences.

The aim of this study was to physiologically compare the two clinical cardiac pacing therapies using objective evaluation indexes. Here, myocardial work was assessed using a visual quantitative evaluation of the pressure-strain ring derived from ultrasonic speckle tracking imaging technology.

The following were the judgment criteria for successful pacing using the left His-Purkinje system: (1) Pacing QRS width significantly narrowed (<120 ms); (2) V1 lead tip unipolar pacing QRS wave in the Qr/QR/qr form; (3) Pacemaker R wave time to peak of V5 lead (pacemaker signal to R wave peak) <80 ms; (4) It was not required from the electrogram that the beam branch potential be seen in the cavity or whether the SV interval (the distance from the stimulus signal to the start of QRS wave). The security range criteria to judge electrode tip depth involved the following: (1) Intraoperative immediate tip unipolar pacing impedance from 700 to 1,200Ω, ring unipolar impedance <tip unipolar impedance or ring unipolar impedance <2,000Ω; (2) Tip monopole pacing threshold <1.0 V/0.4 ms, tip monopole pacing threshold <ring monopole pacing threshold. After meeting these criteria, the C315-His sheath tube was removed with a special incision and the wire was fixed. Before inclusion in the HPSAP study group, the pacing location was verified by a transthoracic echocardiogram (TTE).

Alternatively, the VSP wire implantation process involved placing a 7F sheath through the axillary vein under the guidance of the guide wire and inserting a 58 cm active electrode wire along the sheath to the inferior vena cava. The electrode wire was then adjusted to the right ventricular septum under the support of a plastic inner core wire. The relationship among the wire, tricuspid ring, anterior interventricular groove, and cardiac apex was evaluated through right anterior oblique fluoroscopy. The angular relationship between the end of the wire and the ventricular septum was observed through the left anterior oblique fluoroscopy. After determining the position of the wire under fluoroscopy, the electrode wire screw head was fixed to the right ventricular surface of the interventricular septum, and the plastic inner core wire was extracted. Since there was no obvious clinical difference, no requirement was made for high, middle, and low interventricular septum placement. The test electrode parameter evaluation criteria involved bipolar pacing impedance ranging from 400 to 1,000Ω, bipolar pacing threshold of 1.0 V/0.4 ms or less, bipolar perceptual of 5.0 mV or greater, and the damage current was visible in the far field of the cavity. Afterward, the sheath was removed and the wire was fixed.

## Materials and methods

### Statement

The study was approved by the Institutional Review Board of Sichuan Academy of Medical Sciences, Sichuan Provincial People’s Hospital.

### General information

We retrospectively identified patients who received atrioventricular pacemaker implantation (DDDR pacemaker) in the Cardiovascular Division of our hospital from February 2018 to September 2019. According to the inclusion and exclusion criteria, patients meeting the requirements were screened periluminally, and arrangements were made for echocardiography at our hospital 1 year after implantation.

**FIGURE 1:**
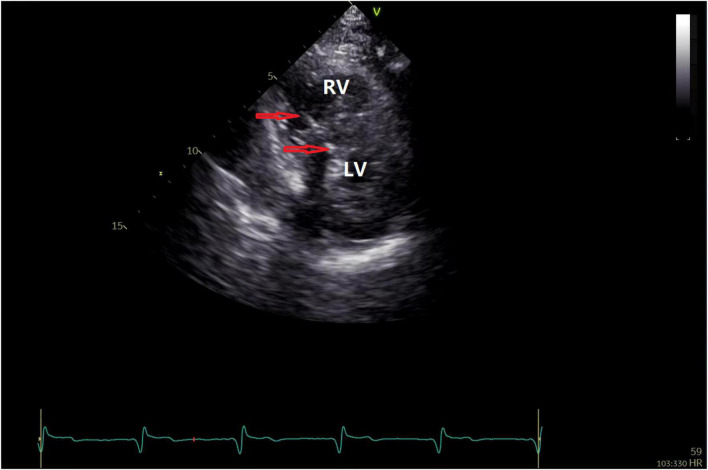
Echocardiography to position the HPSAP electrode. The red arrow indicates that the end of the pacemaker electrode is located below the endocardium of the left ventricle.

Inclusion criteria: Pacemaker implantation in patients with III° AVB. Before pacemaker implantation, LVEF was within the normal range (55% < LVEF < 80%). Exclusion criteria: 1. Complicated arrhythmia; 2. LVEF decreased (LVEF < 55%); 3. Aortic stenosis or moderate or above reflux; and 4. Complicated hypertension, kidney disease, and cardiomyopathy. Additionally, the pacemaker programming method of patients enrolled in the case group should meet the following criteria. (1) Subjects with continuous ventricular pacing dependence after implantation were excluded. Double-chamber permanent pacemaker implantation patients with a preoperative diagnosis of III° AVB were included, and the proportion of ventricular pacing (VP) was confirmed to be greater than 95% between the last follow-up and the date of test by the programmed pacemaker. (2) To ensure that the subject’s ventricular excitement does not bypass its own atrial-ventricular transmission fusion, the programmed pacemaker was set to 350 ms without its own atrial-ventricular transmission after PAV and SAV (the pacemaker was in the state of VP). (3) After the aforementioned verification, to unify the subjects’ AV interphase, the AV interphase was adjusted to PAV/SAV 160/120 (MS). (4) The pacing QRS width of the subjects in ventricular pacing state was measured and recorded manually. The measurement method employed was the lead with the widest QRS wave in the subject’s standard 12-lead electrocardiogram. Before inclusion in the HPSAP group, the pacing position was verified by TTE (Figure 1).

According to the criteria, a total of 43 patients were collected and divided into the following categories according to the method of pacemaker implantation. A total of 20 patients were included in the VSP group, comprising 15 men and 5 women ranging from 52 to 80 years old (mean ± s.d., 68.7 ± 12.3). In the HPSAP group, there were 23 patients, comprising 20 men and 3 women, aged from 55 to 78 years (72.4 ± 4.0). As control group subjects, 40 volunteers with no abnormalities in biochemistry, ECG, and or cardiac ultrasound examination were selected, including 30 men and 10 women, aged from 54 to 77 years (64.3 ± 10.5).

### Operative procedures

For this study, the HPSAP wire implantation process involved inserting a C315-His sheath tube (Medtronic) and 3,830 electrode wire (Medtronic) through the axillary vein. After connecting a polyconductive physiological recorder, His bundle potentials were plotted at the base of the right ventricular septum, and the sheath tube and electrode wires were gradually moved along the line from the H wave to the apex of the heart, reaching the middle and far sections of the His bundle (where the H wave height becomes lower and the HV interval shortens). The QRS waveform of the leading V1 in the right ventricular myocardium was typical of the “W” type. To position the electrode, the angle between the sheath tube and the septum and the length of the electrode sheath tube was adjusted before preliminary testing of the pacing threshold and impedance. The sheath tube was fixed, and the head end of the 3,830 electrode conductor was rotated clockwise to enter the septum under perspective observation. To comprehensively position the screw-in depth, X-ray location from different angles, pacing QRS wave morphology, and pacing impedance measures were used. In some cases, a contrast agent was injected through the sheath tube to observe the electrode screw-in depth.

### Instruments and methods

Blood pressure data were collected from study subjects in a calm state using a cuff and brachial artery pressure measurements of the left arm. The mean value was recorded after three measures. Cardiovascular imaging was performed using a GE Vivid E9 ultrasonic diagnostic instrument fitted with an M5S probe and used at a frequency of 1.5–4.6 MHz, or the 4 V probe at a frequency of 1.5–4.0 MHz. Image analysis was undertaken using the EchoPAC workstation. Conventional synchronous electrocardiogram measurements were taken from patients in the left lateral recumbent position using the M5S probe to evaluate left ventricular function and blood flow at each flap. Switching to the 4 V probe and with patients holding their breath, tri-plane measurements were used to obtain two-dimensional grayer dynamic map storage of the four cavities, three cavities, and two cavities of the standard apex of three cardiac cycles. Images were then collected and stored in the “4D” mode to ensure that the inner and outer membranes of the left ventricle were within the ultrasonic sector and the frame frequency adjusted to more than 40% of the heart rate.

### Image analysis

**FIGURE 2:**
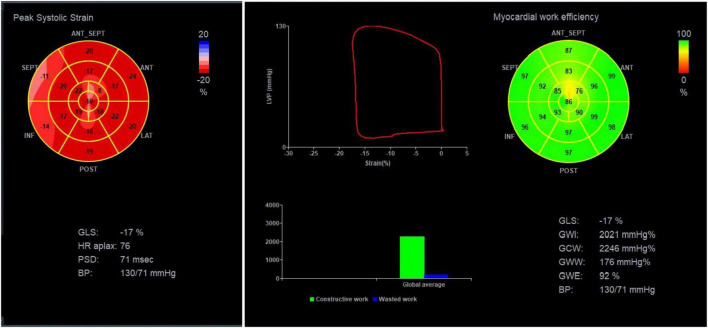
Collection of myocardial work parameters from the EchoPAC workstation. Illustration of the 17-segment strain time curve and calculation of GLS, GWI, GCW, GWW, and GWE.

**FIGURE 3:**
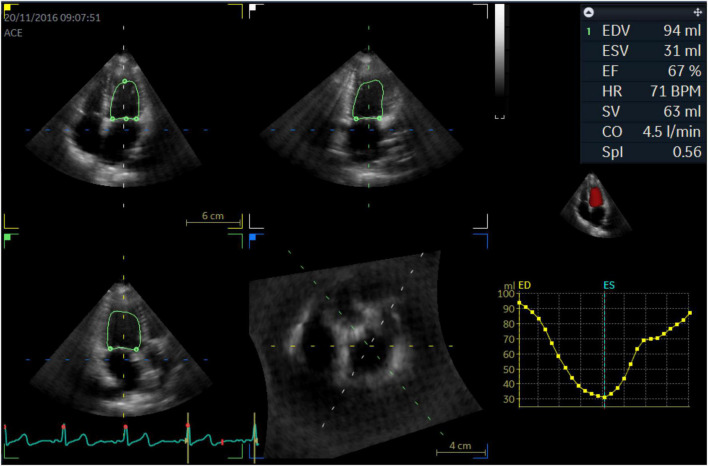
4D mode to obtain EDV, ESV, and SPI parameters.

Images were imported into the EchoPAC workstation (version 203), and the opening and closing time of “AV” and “MV” was determined. Three dynamic grayscale images were selected, and the endocardial surface was drawn automatically or manually. After acquiring the 17-segment strain time curve, myocardial work parameters were calculated, including GWI, GWE, GCW, and GWW of the myocardium at the same cardiac cycle (Figure 2). Other parameters including left ventricular end-diastolic volume, left ventricular end-systolic volume, left ventricular spherical index (SPI), stroke volume, left ventricular ejection fraction, and PSD were obtained from the three-dimensional volume images (Figure 3).

### Statistical analysis

Measurement data conforming to a normal distribution were adopted ($\bar{x}\pm s$). A single-factor variance ANOVA method was used where data conformed to the homogeneity of variance assumption between groups, or if not, the Games-Howell’s multiple comparisons test was used. Nonuniform variance parameters were tested by the rank sum test. SPSS Statistics 22 analysis software was used, with *p* < 0.05 considered to be statistically significant.

## Results

### General comparison of clinical data

Among the patient analysis groups, the body surface area of the HPSAP group was larger than the other two groups, and the left ventricular SPI of patients in the two pacemaker groups was slightly higher than that of the control group (*p* < 0.05). No other statistically significant differences were found for other parameters within groups (Table 1).

**TABLE 1:** Comparison of general data between the normal control, ventricular septal pacing (VSP) and His-Purkinje system area pacing (HPSAP) groups.

	*N*	Age	BSA (kg/m^2^)	SPI (%)	LVEF (%)	EDV (ml)	ESV (ml)	HR	HP (s⋅mmHg)	HP (d⋅mmHg)
VSP	20	69 ± 12	1.63 ± 0.16^Δ^	0.41 ± 0.10*	58.20 ± 5.87	81.90 ± 12.89	34.90 ± 11.41	74 ± 11*^Δ^	137 ± 13	75 ± 13*
HPSAP	23	72 ± 4	1.73 ± 0.09*^Δ^	0.42 ± 0.09*	57.95 ± 4.35	89.26 ± 12.80	36.26 ± 11.24	68 ± 8^Δ^	133 ± 19	76 ± 9*
Normal	40	64 ± 11	1.62 ± 0.15*	0.37 ± 0.06*	61.76 ± 7.16	88.76 ± 19.21	31.88 ± 12.05	67 ± 6*	118 ± 10	64 ± 6*
*P*		0.025	0.167	0.064	0.988	0.624	0.232	0.452	0.242	0.000

BSA, body surface area; SPI, left ventricular sphericity index; LVEF, left ventricular ejection fraction; EDV, left ventricular end-diastolic volume; ESV, left ventricular end-systolic volume; HR, heart rate; HP(S) terminal systolic pressure and HP(D) terminal diastolic pressure.

*Statistically significant differences (*p* < 0.05) between normal control and pacing groups.

^Δ^Statistically significant differences (*p* < 0.05) between the pacing groups.

### Comparison of left ventricular function parameters within groups

**FIGURE 4:**
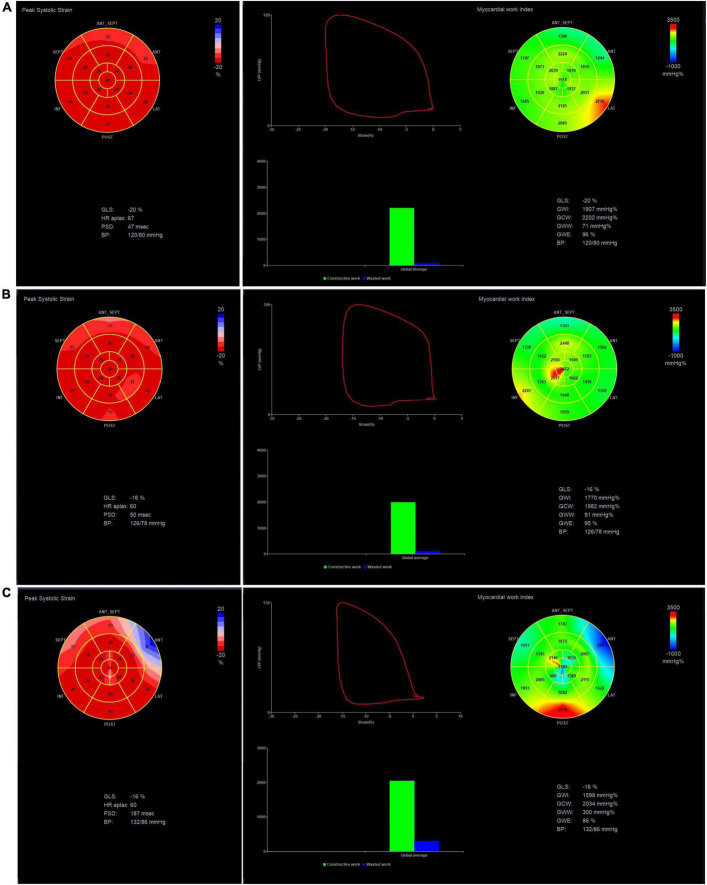
GLS, GWI, GCW, GWW, and GWE of the three groups at 4D mode. **(A)** Normal group, **(B)** HPSAP group, and **(C)** VSP group. Comparing the three groups, the strain bull’s eye graph of the normal group is red, the VSP group is lighter, and even appears blue; the work index bull’s eye graph of the normal group has a uniform green distribution, and the GWI is higher. The work index bull’s eye graph of the VSP group indicated that the work index was unevenly distributed, and the GWI was the lowest.

For GWI measurements along with GWE and GCW, the rank order was the control group > HPSAP group > VSP group (*p* < 0.05), respectively. Whereas for GWW, the findings were VSP group > HPSAP group > control group (*p* < 0.05). Comparing the global and stratified longitudinal strain of the apical four, two, and three chambers, we found the control group > HPSAP group > VSP group (*p* < 0.05; Figure 4 and Table 2).

**TABLE 2:** Comparison of myocardial work and myocardial stratification strain among the three groups.

	4D-GLS (%)	GWI (mmHg%)	GWE (%)	GCW (mmHg%)	GWW (mmHg%)	PSD	QRS (ms)
HPSAP	−15.21 ± 3.63*	1959.30 ± 601.37*	92.56 ± 4.94*	1993.32 ± 296.87	157.30 ± 54.84*	67.69 ± 20.88*	123 ± 18.50
SVP	−10.95 ± 2.30*	1552.55 ± 329.29*	83.90 ± 4.03*	1846.45 ± 322.37	324.20 ± 97.09*	78.05 ± 20.91*	>350 ms
Normal	−16.60 ± 4.26	1982.96 ± 239.50	96.40 ± 1.73	2184.17 ± 577.13	51.52 ± 14.43	34.80 ± 6.99	93 ± 6.48 ms
H	13.787	7.900	32.800	2.033	32.726	31.978	33.228
*P*	0.001	0.002	0.000	0.286	0.000	0.000	0.000

4D-GLS,four-dimensional global longitudinal strain; GWI, global myocardial work index; GWE, global myocardial work efficient; GCW, global myocardial constructive work; GWW, global myocardial wasted work; PSD, peak strain dispersion.

*Statistically significant differences (*p* < 0.05) between normal control and pacing groups.

Other comparisons among the three groups showed that compared with the control group, the GCW of the left ventricular segmental myocardium (17/17 segments) in the VSP group was significantly reduced (*p* < 0.05), while 70.59% (12/17 segments) of the HPSAP group was lower than the control group (*p* < 0.05). GCW measures in 29.41% (5/17 segments) of the HPSAP group were bigger than the VSP group (*p* < 0.05) and mainly concentrated in the ventricular septum and inferior wall (Table 3).

**TABLE 3:** Comparison of myocardial constructive work among the three groups at 17 segments of the left ventricle ($\bar{x}\pm s$, %).

	Segment	HPSAP	VSP	Normal
	Posterventricular septum	85.26 ± 12.55*	86.00 ± 8.99*	96.36 ± 2.65
	Interventricular septum	87.73 ± 9.32*	85.20 ± 7.83*	96.52 ± 1.93
Left ventricular basement level	Anterior wall	91.43 ± 2.99*	85.75 ± 10.17*	96.68 ± 1.70
	Lateral wall	89.13 ± 12.75	87.50 ± 11.56*	95.40 ± 2.67
	Posterior wall	90.56 ± 7.97	87.60 ± 10.29*	94.84 ± 3.33
	Inferior wall	93.47 ± 5.75^Δ^	82.30 ± 16.80*^Δ^	93.96 ± 4.04
	Posterventricular septum	91.13 ± 11.54*^Δ^	80.85 ± 14.43*^Δ^	98.32 ± 2.94
	Interventricular septum	95.17 ± 5.60^Δ^	85.80 ± 5.89*^Δ^	97.88 ± 2.00
Left ventricular middle level	Anterior wall	94.26 ± 4.36*	90.40 ± 6.99*	97.00 ± 2.30
	Lateral wall	94.13 ± 4.99*	91.65 ± 6.08*	97.60 ± 1.82
	Posterior wall	94.21 ± 7.24	86.95 ± 11.72*	97.28 ± 2.63
	Inferior wall	95.00 ± 4.89^Δ^	84.65 ± 13.95*^Δ^	96.88 ± 1.83
	Apical septum	89.39 ± 10.16*^Δ^	73.00 ± 21.73*^Δ^	96.52 ± 3.59
Left ventricular apex level	Apical-anterior wall	86.26 ± 13.44*	83.90 ± 6.52*	96.96 ± 2.71
	Apical-lateral wall	89.26 ± 8.92*	82.85 ± 15.14*	95.96 ± 3.61
	Apical-inferior wall	86.34 ± 13.65*	81.75 ± 8.46*	97.08 ± 2.76
	Apical cap	87.82 ± 11.28*	81.15 ± 9.68*	96.32 ± 2.56

*Statistically significant differences (*p* < 0.05) between normal control and pacing groups. ^Δ^Statistically significant differences (*p* < 0.05) between the pacing groups.

## Discussion

Cardiac resynchronization therapy (CRT) using His bundle pacing can reproduce real physiological pacing in patients (5, 6). Although the success rate of this surgical approach has been greatly improved through both technological innovation and methodological improvements (7, 8), there are still deficiencies to overcome, including high threshold, low perception, and fixation difficulty (9, 10). Different from the small structure of the Schiff beam, the His-Purkinje system involves a wide and interconnected conduction system structure under the left endocardium and is easier to be anatomically located (11), and the surgical success rate is higher (12). Moreover, compared with Hipper bundle pacing, the His-Purkinje system area pacing across the bundle block was reported to be more advantageous in patients with atrioventricular block or left bundle branch block (13). Regarding the latter, the left fasciculus has abundant myocardial tissue around it, which was more perceptive and maneuverable (3, 14).

In 2017, Huang Jianwei’s team described the left bundle branch pacing technology under the intima of the left ventricle (15), which produced stable and low pacing thresholds and good ventricular perception on the basis of ensuring left ventricle synchronization. Subsequent studies have shown that the left bundle-branch pacing technique has a high success rate of implantation, which proved helpful in improving left ventricular systolic function (16–19). Currently, all clinical reports on this pacing pattern are based on follow-up observations, but there are few reports both in China and abroad evaluating this pacing pattern using advanced ultrasound technology (20).

Here, the pressure-strain ring parameter proposed by Russell et al. (21) was used to non-invasively estimate left ventricular pressure by measuring cuff blood pressure and setting valve opening time. This approach has been validated in a variety of pathologic disorders, excluding severe stenosis and reflux. The normalization of the pressure curve was achieved by synthesizing invasive pressure measurements from patients with many different pathological conditions and standardizing the isovolumic systolic, ejection, and isovolumic diastolic and peak pressures into equal durations. Pressure-volumetric area is defined as the myocardial work done by the myocardium per stroke (ignoring energy loss). The work done by each cardiac segment can be expressed as the area of the strain-pressure ring (Figure 2). Since the left ventricular pressure cannot be completely divided into the force generated by each segment, the myocardial work done by each segment is simplified to represent the left ventricular stroke work.

Other scholars (22) have reported an ultrasonic speckle tracking technology derivative of a left ventricular pressure-strain ring as a noninvasive evaluation method for quantifying regional myocardial function. Indeed, they found this approach can also provide information about metabolic needs since there was a strong correlation and consistency between the noninvasive left ventricular pressure-strain ring area measurements and findings using FDG-PET local glucose metabolism. Glucose metabolism affects myocardial work, and the area of the pressure-strain ring can reflect the difference in myocardial work distribution. The left ventricular pressure-strain ring integrates the measurement of myocardial deformation and left ventricular pressure, providing a new method for quantifying myocardial work with potential advantages over traditional global longitudinal strain.

Work distribution evaluation allows clinicians to better evaluate ischemic patients and select CRT candidates. Furthermore, it provides valuable additional information on left ventricular function in patients after CRT (23, 24). In addition, MW was concluded to be better than strain in evaluating cardiac function considering deformation and afterload, highlighting the myocardial work index as a promising tool in CRT patient management. On this basis, our study applied this technology to preliminarily evaluate HPSAP and VSP to explore the difference in left ventricular function under the two different pacing modes.

A previous report by Mafi-Rad et al. (25) found that compared with right ventricular apical or septal pacing, left ventricular septal pacing produced a narrower QRS duration and improved the acute hemodynamic effect. Our study confirmed that the regional pacing of the His-Purkinje system can realize left ventricular resynchronization, with a low threshold and stable, good perception, and significantly improved cardiac function of patients, which can be used as a supplement of the His bundle pacing. The His-Purkinje system area pacing is also expected to be an alternative to biventricular pacing (26). Our results showed that the PSD of each segment of the left ventricle in HPSAP was significantly lower than after VSP. Moreover, the characteristics of the regional pacing of the His-Purkinje system are closer to physiological electrical conduction; the left ventricle has better directivity, coordination, and effectiveness in each segment of the myocardium, making a more coordinated and effective contraction of the left ventricular myocardium. Additionally, we found no differences in LVEF and no left ventricular segmental ischemia between patient groups, although the GWI and GCW of HPSAP patients were significantly higher than for VSP. Studies have shown that VSP is theoretically closer to physiological electrical stimulation, which can significantly shorten QRS waves, but it can also cause a certain degree of ventricular myocardium contractile asynchronism (27, 28). Compared with patients with HPSAP, left ventricular inactivity increased in patients with VSP. The likely explanation for this difference is that the left ventricular segmental myocardium in VSP cannot work in a coordinated and efficient manner, resulting in high “energy loss” and useless effort in some myocardium, which is consistent with our previous findings (29).

Finally, we must consider some of the limitations and future improvements of our study. Although there were no gender or age-related changes among the patient groups, there were differences in diastolic blood pressure. However, on the basis of reported findings by Russell et al., only GWI and GCW are significantly associated with systolic blood pressure. Other considerations include the following: (1) Although all enrolled patients with III° AVB were implanted with pacemakers, their health was complicated by various diseases, with a small number of patients with hypertension; (2) Further confirmation of our findings is warranted through increasing the sample size and refining the patient groups studied; (3) Image quality effects may potentially affect the research results; and (4) The follow-up time for studying the His-Purkinje system area pacing was limited.

## Conclusion

This study confirms that CRT with HPSAP enables the left ventricular myocardium to work more harmonically and efficiently than VSP, at least within 1 year of follow-up. We plan to use the new ultrasound technology more extensively by enrolling more pacing cases and extending the follow-up to the medium and long term. Increasing the sample size and observation period will provide a more objective basis for selecting clinical pacing treatments.

## Data availability statement

The raw data supporting the conclusions of this article will be made available by the authors, without undue reservation.

## Ethics statement

The studies involving human participants were reviewed and approved by Sichuan Provincial People’s Hospital Ethics Committee. The patients/participants provided their written informed consent to participate in this study.

## Author contributions

YL, YT, and QM contributed to the conception of the study. QM and YL performed the experiment. YD and CL contributed significantly to analysis and manuscript preparation. QM and SW performed the data analyses and wrote the manuscript. TF, HX, JL, XL, and ZG helped perform the analysis with constructive discussions. All authors contributed to the article and approved the submitted version.
